# ATP Sulfurylase is Essential for the Utilization of Sulfamate as a Sulfur Source in the Yeast *Komagataella pastoris* (syn. *Pichia pastoris*)

**DOI:** 10.1007/s00284-017-1276-0

**Published:** 2017-06-12

**Authors:** Tomas Linder

**Affiliations:** Department of Molecular Sciences, Swedish University of Agricultural Sciences, Box 7015, 750 07 Uppsala, Sweden

## Abstract

The methylotrophic yeast *Komagataella pastoris* (syn. *Pichia pastoris*) is one of the few known yeasts that can utilize sulfamate (${\text{NH}}_{2} {\text{SO}}_{3}^{ - }$) as a sulfur source. The biochemical pathway responsible for the catabolism of sulfamate has yet to be identified. The present study sought to investigate whether sulfamate catabolism proceeds through either of the inorganic sulfur intermediates sulfate (${\text{SO}}_{4}^{2 - }$) or sulfite (${\text{SO}}_{3}^{2 - }$) before its assimilation and subsequent incorporation into sulfur-containing amino acids and their derivatives. Two key genes in the *K. pastoris* inorganic sulfur assimilation pathway were deleted separately and the ability of each deletion mutant to utilize sulfamate and other selected sulfur sources was studied. Deletion of the *MET3* gene (which encodes the enzyme ATP sulfurylase) did not affect growth on l-methionine, sulfite, methanesulfonate, or taurine but completely abolished growth on sulfate, methyl sulfate and sulfamate. Deletion of the *MET5* gene (which encodes the β subunit of the enzyme sulfite reductase) abolished growth on all tested sulfur sources except l-methionine. These results suggest that the catabolism of sulfamate proceeds through a sulfate intermediate before its assimilation.

## Introduction

**Fig. 1:**
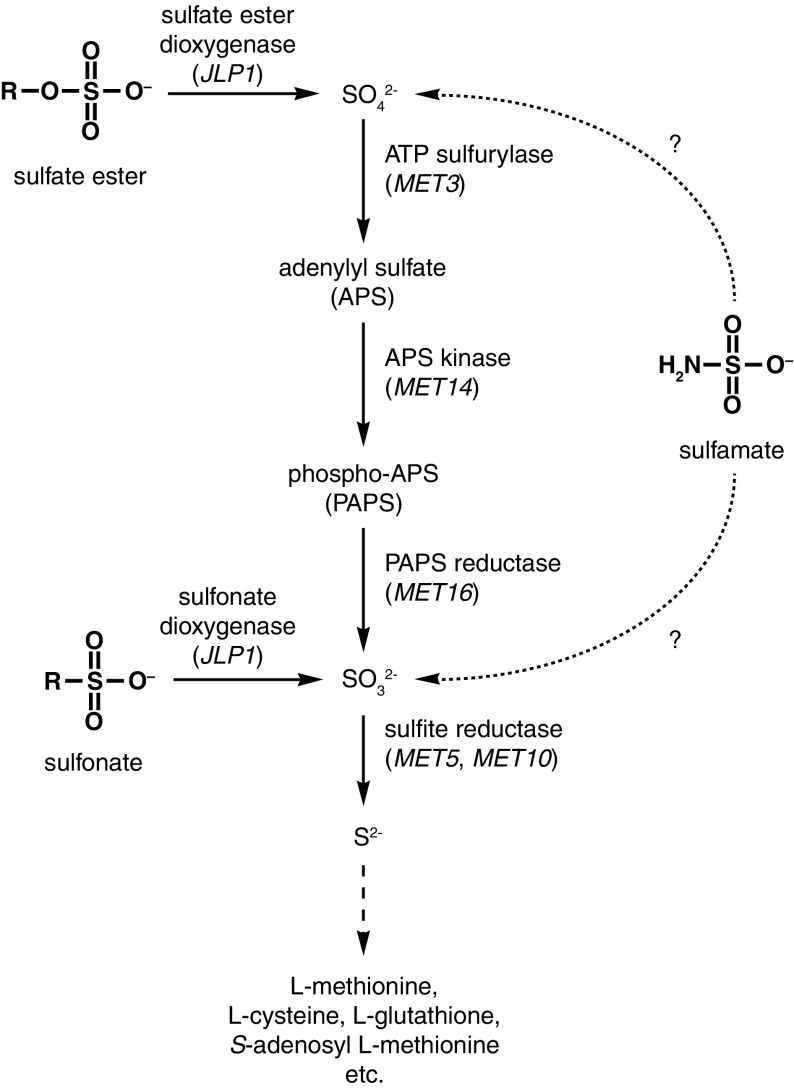
A simplified overview of the inorganic sulfur assimilation pathway in budding yeasts such as *S. cerevisiae* and *K. pastoris*. Gene names of the corresponding enzymes are shown in *brackets*

Sulfur is an essential element for cellular function and is found in a wide variety of organic biomolecules including the proteinogenic amino acids methionine and cysteine, the redox regulator glutathione, the methylation donor *S*-adenosyl methionine and the cofactors biotin, coenzyme A, lipoic acid, and thiamine. The majority of plants and microorganisms can assimilate inorganic forms of sulfur such as sulfate (${\text{SO}}_{4}^{2 - }$), sulfite (${\text{SO}}_{3}^{2 - }$), and sulfide (S^2−^) from their external environment. The conventional inorganic sulfur assimilation pathway is broadly conserved between plants, fungi, bacteria, and archaea with some minor variations between kingdoms [2, 9, 12, 13]. Inorganic sulfur assimilation in yeast and other fungi involves the conversion of sulfate into sulfide via sulfite in four enzymatic steps (Fig. 1). In the first step, intracellular sulfate becomes activated through conjugation to AMP by the enzyme ATP sulfurylase (encoded by the *MET3* gene) to form adenylyl sulfate (APS). APS is then converted into phospho-APS (PAPS) by APS kinase (encoded by the *MET14* gene). PAPS is subsequently reduced by the enzyme PAPS reductase (encoded by the *MET16* gene) to release sulfite. The sulfite that is released from PAPS is reduced into sulfide by sulfite reductase (encoded by the *MET5* and *MET10* genes). Sulfide can then be incorporated into amino acid pre-cursors and that are subsequently converted into various sulfur-containing biomolecules.

Many microorganisms can also assimilate non-amino acid organosulfur compounds such as sulfoxides, sulfones, sulfonates, and sulfate esters [5, 14], which are abundant in nature [1, 10]. These compounds are first desulfurized to release inorganic sulfur, usually in the form of either sulfate or sulfite [3–5, 14], which can then be assimilated through the inorganic sulfur assimilation pathway. In the common Baker’s yeast *Saccharomyces cerevisiae*, the *JLP1* gene, which encodes an α-ketoglutarate-dependent dioxygenase, is responsible for the desulfurization of sulfonates [3] and possibly sulfate esters as well (Fig. 1).

The yeast *Komagataella pastoris* (syn. *Pichia pastoris*) is one of a few species of yeast that are known to utilize sulfamate (${\text{NH}}_{2} {\text{SO}}_{3}^{ - }$) as a sulfur source [5]. Nothing is currently known about the catabolic pathway for sulfamate in yeasts but it is likely to involve the cleavage of the N–S bond to release either sulfate or sulfite (Fig. 1). The present study sought to investigate whether the inorganic sulfur assimilation pathway is required for the utilization of sulfamate and if so, identify the inorganic sulfur intermediate in *K. pastoris* sulfamate assimilation. This was accomplished through individual deletion of two key genes in the inorganic sulfur assimilation pathway—*MET3* and *MET5*. Phenotypical analysis of both mutants on sulfamate as well as selected reference sulfur compounds was then used to identify the likely inorganic sulfur intermediate.

## Materials and Methods

### Yeast Integration Constructs

**Fig. 2:**
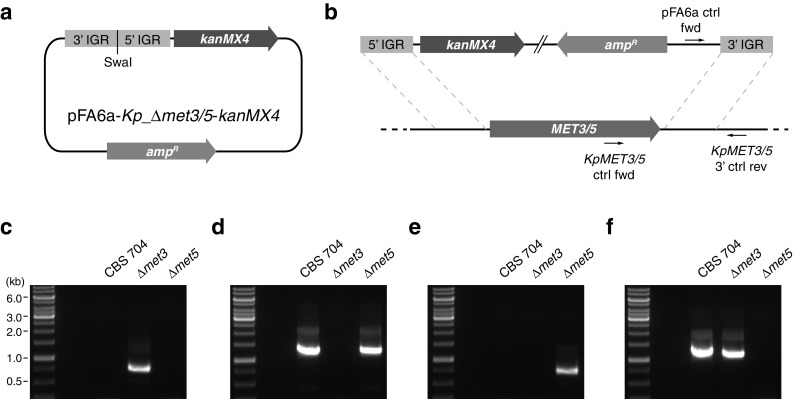
Plasmid design and integration of plasmid constructs at the *K. pastoris MET3* and *MET5* loci. **a** Design of the *MET3* and *MET5* integration plasmids. The *Kp_Δmet3* and *Kp_Δmet5* targeting cassettes were assembled in the pFA6a-*kanMX4* plasmid to produce plasmids pFA6a-*Kp_Δmet3*-*kanMX4* and pFA6a-*Kp_Δmet5*-*kanMX4*, respectively. Plasmid components are not drawn to scale. **b** The plasmids pFA6a-*Kp_Δmet3*-*kanMX4* and pFA6a-*Kp_Δmet5*-*kanMX4* were linearized by digestion with *Swa*I to enable homologous recombination with the 5′ and 3′ IGRs of *MET3* and *MET5*. The locations of control primers to confirm correct integration of the construct are indicated. DNA elements are not drawn to scale. **c** Confirmation of the correct integration of the linearized pFA6a-*Kp_Δmet3*-*kanMX4* construct as demonstrated by PCR of genomic DNA using primers pFA6a fwd and *KpMET3* 3′ ctrl rev. **d** Confirmation of the removal of the endogenous *MET3* locus as demonstrated by PCR of genomic DNA using primers *KpMET3* ctrl fwd and *KpMET3* 3′ ctrl rev. **e** Confirmation of the correct integration of the linearized pFA6a-*Kp_Δmet5*-*kanMX4* construct as demonstrated by PCR of genomic DNA using primers pFA6a fwd and *KpMET5* 3′ ctrl rev. **f** Confirmation of the removal of the endogenous *MET5* locus as demonstrated by PCR of genomic DNA using primers *KpMET5* ctrl fwd and *KpMET5* 3′ ctrl rev

The intergenic regions (IGRs) flanking the *K. pastoris MET3* and *MET5* ORFs were amplified and inserted into the pFA6a-*kanMX4* plasmid (GenBank accession AJ002680, [15]) to enable targeted gene replacement. The *KpMET3* 5′ IGR (GenBank accession CABH01000262, residues 20,406–20,990) was amplified from *K. pastoris* CBS 704 genomic DNA with primers *KpMET3* 5′ fwd (GCG CGC CCC GGG ATT TAA ATG CCG AAA GAT TCA A) and *KpMET3* 5′ rev (GCG CGC GGA TCC TTG AGA GAT CTT TCA CTG), digested with *Bam*HI and *Sma*I, and inserted into *Bgl*II/*Sma*I-cut pFA6a-*kanMX4* to produce the intermediate plasmid pFA6a-*KpMET3*_5′-*kanMX4*. The *KpMET3* 3′ IGR (GenBank accession CABH01000262, residues 18,241–18,734) was amplified from *K. pastoris* CBS 704 genomic DNA with primers *KpMET3* 3′ fwd (GCG CGC GGA TCC ATA GTA GAC TTT GTA ATG) and *KpMET3* 3′ rev (GCG CGC ATT TAA ATA ATG AGT ATG TTA TC), digested with *Bam*HI and *Swa*I and inserted into *Bam*HI/*Swa*I-cut pFA6a-*KpMET3*_5′-*kanMX4* to produce the plasmid pFA6a-*Kp_Δmet3*-*kanMX4* (Fig. 2a).

The *KpMET5* 5′ IGR (GenBank accession CABH01000235, residues 383,892–384,453) was amplified from *K. pastoris* CBS 704 genomic DNA with primers *KpMET5* 5′ fwd (GCG CGC CCC GGG ATT TAA ATT ATG GAT TAT GTA A) and *KpMET5* 5′ rev (GCG CGC AGA TCT TCA ACC AAC AGA ACC GGT CAT), digested with *Bgl*II and *Sma*I and inserted into *Bgl*II/*Sma*I-cut pFA6a-*kanMX4* to produce the intermediate plasmid pFA6a-*KpMET5*_5′-*kanMX4*. The *KpMET5* 3′ IGR (GenBank accession CABH01000235, residues 379,251–379,682) was amplified from *K. pastoris* CBS 704 genomic DNA with primers *KpMET5* 3′ fwd (GCG CGC GGA TCC GAA GAA GCC ATT GCA TAG) and *KpMET5* 3′ rev (GCG CGC ATT TAA ATG TAA CGT AAC CTT A), digested with *Bam*HI and *Swa*I and inserted into *Bam*HI/*Swa*I-cut pFA6a-*KpMET5*_5′-*kanMX4* to produce the plasmid pFA6a-*Kp_Δmet5*-*kanMX4* (Fig. 2a). Prior to transformation the pFA6a-*Kp_Δmet3*-*kanMX4* and pFA6a-*Kp_Δmet5*-*kanMX4* plasmids were linearized through digestion with *Swa*I (Fig. 2b) and purified into sterile water using the QIAquick PCR purification kit (Qiagen).

### Yeast Transformation

The parent yeast strain for the transformations in this study was *K. pastoris* CBS 704, which was purchased from Centraalbureau voor Schimmelcultures (Utrecht, the Netherlands). The selection agent G418 disulfate was purchased from Formedium Ltd. (Norfolk, UK). An aqueous stock solution of G418 disulfate was prepared to a final concentration of 100 g l^−1^, sterilized by filtration and stored as aliquots at −20 °C. The transformation protocol used in this study has been described previously [6]. Following transformation, cells were plated out on selective media consisting of YMPD agar (3 g yeast extract l^−1^, 3 g malt extract l^−1^, 5 g peptone l^−1^, 10 g glucose l^−1^, 20 g agar l^−1^) with 400 mg G418 disulfate l^−1^. Correct chromosomal integration and deletion of the *MET3* and *MET5* loci were confirmed by PCR analysis of purified genomic DNA from each strain (Fig. 2c–f). Correct integration at the *MET3* locus was assayed using primers pFA6a ctrl fwd (ACT GAG AGT GCA CCA TAT GGA) and *KpMET3* 3′ ctrl rev (TCG GTT GTC AGA TGG CA), which produce no product in the CBS 704 parent strain and the *Δmet5* strain but a 715-bp amplification product in the *Δmet3* strain (Fig. 2c). Successful deletion of the *MET3* gene was assayed using primers *KpMET3* ctrl fwd (TAC GCT CCA ATT GAC ACA GTC) and *KpMET3* 3′ ctrl rev, which produce a 1199-bp amplification product in the CBS 704 parent strain and the *Δmet5* strain but no product in the *Δmet3* strain (Fig. 2d). Correct integration at the *MET5* locus was assayed using primers pFA6a ctrl fwd and *KpMET5* 3′ ctrl rev (TCT CGA GAC ACA CAT CTG), which produce no product in the CBS 704 parent strain and the *Δmet3* strain but a 643-bp amplification product in the *Δmet5* strain (Fig. 2e). Successful deletion of the *MET5* gene was assayed using primers *KpMET5* ctrl fwd (TCA TAA GAC TGG TCT CCT GGA) and *KpMET5* 3′ ctrl rev, which produce a 1107-bp amplification product in the CBS 704 parent strain and the *Δmet3* strain but no product in the *Δmet5* strain (Fig. 2f).

### Sulfur Utilization Assays

A sulfur-limited glucose medium (SLD) with only trace amounts of sulfate (≤6 μM) was used for assaying growth on individual sulfur-containing compounds [5]. SLD medium consisted of 1.2 g yeast nitrogen base without amino acids, ammonium sulfate, or magnesium l^−1^ (Formedium Ltd., Norfolk, UK), 4 g ammonium chloride l^−1^, 0.84 g magnesium chloride hexahydrate l^−1^, and 20 g glucose l^−1^. Prior to the sulfur utilization assay, each yeast strain was pre-cultured in 3 ml methionine-supplemented minimal glucose medium consisting of 6.7 g Difco yeast nitrogen base without amino acids l^−1^ (Becton, Dickinson and Company), 20 g glucose l^−1^, and 1 mM l-methionine. Pre-cultures were washed twice in sterile deionized water before being re-suspended in 2.97 ml SLD in a 50-ml tube to a final optical density at 600 nm (OD_600_) of 0.005. Individual sulfur-containing compounds were added as 30 μl of a 10 mM stock solution, making a final concentration of 0.1 mM. A non-supplemented sample with 30 μl deionized water was used as a control. Chloramphenicol (15 mg l^−1^) was included to prevent bacterial contamination. Incubations were carried out at 30 °C in a rotary shaker set to 200 rpm. Growth was monitored by measurement of OD_600_ after 6 days using an Ultrospec 1100 pro spectrophotometer with a 1 cm pathlength (GE Healthcare). Each sulfur utilization assay was carried out in triplicate.

## Results and Discussion

The inorganic sulfur assimilation pathway is required both for the assimilation of inorganic sulfur as well as non-amino acid organosulfur compounds such as sulfonates and sulfate esters (Fig. 1). Desulfurization of sulfonates and sulfate esters has not been previously studied in *K. pastoris* but its genome contains two genes (*PIPA01971* and *PIPA02153*) that encode putative α-ketoglutarate-dependent sulfonate/sulfate ester dioxygenases homologous to the *S. cerevisiae JLP1* gene [3] and the bacterial *atsK* gene [4].

ATP sulfurylase (encoded by the gene *MET3*) and sulfite reductase (encoded by the genes *MET5* and *MET10*) are key enzymes in the inorganic sulfur assimilation pathway and deletion of either gene can demonstrate whether the assimilation of a particular sulfur compound proceeds through a sulfate or sulfite intermediate. Deletion of *MET3* is expected to prevent growth on sulfur sources assimilated through a sulfate intermediate (such as sulfate esters) but not those assimilated through a sulfite intermediate (such as sulfonates), while deletion of *MET5* is expected to prevent growth on sulfur sources assimilated through either a sulfate or sulfite intermediate. The present study therefore sought to gain a better understanding of sulfamate catabolism through phenotypical assays of *MET3* and *MET5* deletion mutants cultivated on minimal medium containing a single sulfur source. In addition to sulfamate, six additional sulfur sources were included for reference. These included the sodium salts of sulfate and sulfite, the sulfate ester methylsulfate and the two sulfonates taurine and methanesulfonate. l-Methionine was included as a reference as its assimilation does not require the inorganic sulfur assimilation pathway.

The *K. pastoris MET3* and *MET5* genes were deleted individually by homologous gene replacement using the *kanMX4* G418 disulfate resistance marker (Fig. 2b). Neither mutant displayed any detectable growth defect on rich YMPD medium but failed to show any detectable growth on a minimal medium where sulfate was the only available sulfur source (data not shown). Growth was restored in both mutants if the minimal medium was supplemented with l-methionine. Both mutants were subsequently assayed for growth on chemically defined sulfur-limited medium (SLD) supplemented with different organic or inorganic sources of sulfur to final concentration of 0.1 mM total sulfur. (Neither mutant grew in SLD medium lacking sulfur supplementation.) Growth was measured 6 days after inoculation. The parent strain CBS 704 was included as a reference.

**Fig. 3:**
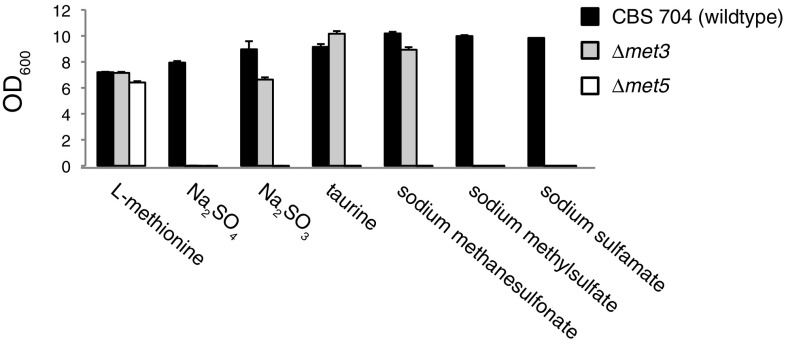
Growth of *K. pastoris* deletion mutants on selected sulfur sources. *Bars* represent the average optical density (OD_600_) value after 6 days incubation in 3 ml chemically defined medium containing 0.1 mM of the indicated sulfur source. Growth assays were carried out in triplicate with *error bars* indicating one standard deviation

As expected neither mutant displayed any detectable growth on sulfate as the only sulfur source after 6 days, while only the *Δmet3* mutant could grow when sulfite was the only available sulfur source (Fig. 3). Both mutants grew well on l-methionine comparable to the CBS 704 parent strain. The *MET3* gene appeared to be entirely dispensable for growth when l-methionine, taurine, or methanesulfonate was the only available sulfur source, which was expected as the assimilation of these sulfur sources does not involve a sulfate intermediate. The *Δmet3* strain did not grow on methyl sulfate, which agrees with previous work that has shown that the assimilation of sulfate esters proceeds through a sulfate intermediate [4]. Notably, neither mutant could grow when sulfamate was the only available sulfur source, which demonstrates the requirement for the inorganic sulfur assimilation pathway in sulfamate assimilation and also suggests that sulfamate assimilation in *K. pastoris* proceeds through a sulfate intermediate.

Deletion of the *MET5* gene abolished growth for all sulfur sources with the exception of l-methionine (Fig. 3). This result was expected for sulfur sources with an absolute requirement for the *MET3* gene (which will consequently not grow in the absence of *MET5* gene either) as well as sulfur compounds known or predicted to be assimilated through a sulfite intermediate such as sulfonates (taurine and methanesulfonate).

In summary, this study has demonstrated for the first time the requirement for the *MET3* gene for the utilization of sulfamate as a sole source of sulfur in yeast. This observation suggests that the catabolism of sulfamate produces sulfate rather than sulfite as an intermediate before assimilation in *K. pastoris* and possibly other sulfamate-utilizing yeasts as well. The enzymatic activity responsible for sulfamate catabolism in *K. pastoris* still remains to be identified. Sulfamate compounds are known to occur in nature, the most predominant form being *N*-sulfated glucosamine residues in the linear glycosaminoglycans heparin and heparan sulfate. Sulfamate functional groups have also been described in a number of natural products [7]. Two enzymes belonging to the arylsulfatase protein family (Pfam accession number PF00884) from human [11] and the bacterium *Pedobacter heparinus* [8] have been shown to desulfurize sulfamate groups in heparan sulfate. Genes encoding proteins belonging to the arylsulfatase family are present in a number of yeasts, including well-characterized species such as *Kluyveromyces lactis*, *Schizosaccharomyces pombe*, and *Yarrowia lipolytica*. However, previous studies have shown no correlation between the presence of putative arylsulfatases in these species and the ability to utilize sulfamate as a sulfur source [5]. The *K. pastoris* CBS 704 genome lacks any detectable arylsulfatase homologs, which further argues against a role for this protein family in sulfamatase utilization in yeast. The significant evolutionary distance between *K. pastoris* and *S. cerevisiae* as well as the greater metabolic versatility of *K. pastoris* with respect to sulfur makes this yeast a promising system for greater understanding of the metabolic pathways, involved in the assimilation of alternative sulfur sources in yeast.
